# Highlights of basic and clinical pharmacology from Africa

**DOI:** 10.1002/prp2.1069

**Published:** 2023-02-27

**Authors:** Vanessa Steenkamp

**Affiliations:** ^1^ Department of Pharmacology, Faculty of Health Sciences University of Pretoria Pretoria South Africa

The special themed issue; ‘Highlights of Basic and Clinical Pharmacology from Africa’ is a compilation of contributions from both basic and clinical research from the African continent. The publications included in this editorial highlight the current status of pharmacology in Africa, a growing topic. This special issue touched on various diseases, as well as topics that show both how diverse pharmacology research in Africa is and how resource‐constrained jurisdictions can manage key medicine issues. The special issue consists of 25 articles focussing on various diseases from atopic dermatitis to sickle cell disease, protection of organ toxicity, adverse drug reactions and off‐label drug use. Contributions were received from diverse countries and although many articles focussed on the situation in a specific country, findings in the sub‐Saharan African region are also available.

It is well known globally that inappropriate use of antibiotics contributes to antimicrobial resistance. To combat this antimicrobial stewardship has been a key focus in recent years, where programs and interventions have been developed with the aim to optimize antimicrobial use. Ayele et al. evaluated the appropriate use of ceftriaxone in medical and emergency wards of Gondar University Referral Hospital in North‐West Ethiopia. Ceftriaxone is one of the most used antibiotics globally due to its high potency, wide spectrum of activity and low risk of toxicity.^1^ The authors assessed the use of ceftriaxone through a cross‐sectional study, analyzing medical records and following standard treatment protocols provided in literature, by the World Health Organization, American Society of Health System Pharmacist's Criteria for Drug Use Evaluation and the Ethiopian Standard Treatment Guidelines. The results from the study confirmed that there is a high and inappropriate use of ceftriaxone which is ultimately harmful to patients.

Birhanu et al. conducted a literature review on the contribution of nano‐based delivery approaches in overcoming ceftriaxone resistance. Ceftriaxone is an antibiotic commonly prescribed due to its broad‐spectrum activity and low toxicity. It was noted that resistance to ceftriaxone has increased significantly, causing challenges in the global healthcare system. The authors reported that several strategies have been put in place to control this, one being a nano‐based delivery system.

Adejumo et al. conducted phenotyping studies of CYP2C19, a genetically polymorphic drug‐metabolizing enzyme^2^ using comparative metabolism of proguanil in sickle‐cell disease patients and healthy controls. Proguanil is an antimalarial drug that can be metabolized by CYP2C19. Following the administration of proguanil, its metabolites and major CYP2C19‐dependent metabolites were measured by high‐performance liquid chromatography. This allowed for the determination of the metabolic ratios which was used to classify subjects as either phenotypically poor or extensive metabolizers. The latter is crucial in determining the dosage regime of proguanil.

The research article of Alijore et al. addresses the benefits of magnesium ion (as MgCl_2_) in organophosphorus (chlorpyrifos) poisoning. The use of pesticides often leads to intentional and intentional poisoning which is a major public health concern, still relatively commonly seen in African medical practice. The authors conducted in vitro investigations to evaluate the benefits of magnesium ions in organophosphorus poisoning since it is known to interact with substrates and membrane enzymes. The findings from the study suggest that MgCl_2_ was able to counteract the effects of the pesticide poisoning by promoting normal ATPase activity and inhibiting the release of acetylcholine from the cell.

Two papers on protective effects of compounds against organ toxicity are included in this issue. Akindele et al. conducted in vitro and in vivo studies to determine whether carvedilol has protective effects on its own as well as in combination with diltiazem and prednisolone against liver and kidney toxicities induced by known nephrotoxic agents (doxorubicin and 5‐FU). Biochemical analysis, antioxidant analysis and histopathological analysis were carried out to gather evidence and correlate the results. Due to the in vivo antioxidant activity of carvedilol, it was noted to protect against doxorubicin and 5‐fluorouracil‐induced hepatic and renal toxicities. The same was observed when carvedilol was administered with diltiazem. However, co‐administration of carvedilol with diltiazem or prednisolone did not offer better protection against toxicity.

Wanas et al. evaluated the protective effects of tolvaptan in cyclophosphamide (CP)‐induced nephrotoxicity. CP is a chemotherapeutic used to treat several diseases such as breast cancer, multiple sclerosis, and lymphomas. This chemotherapeutic agent is also known to be nephrotoxic and this was confirmed in the study as noted by an increase in urea, creatinine, and potassium concentrations. The histopathological changes further correlated with the biochemical results. The co‐administration of CP with tolvaptan was found to provide nephroprotective effects, as indicated by a decrease in the serum urea, creatinine, and potassium levels.

In 2014, the isoniazid preventative therapy (IPT) policy was introduced in Eritrea; this was met with an increase in the number of reports to the Eritrean Pharmacovigilance Centre due to hepatoxicity and fatality. Russom et al. set out to evaluate the association between IPT and hepatotoxicity and identify the risk factors in patients on HAART. It was confirmed that IPT was associated with hepatoxicity, supported by the fact that cases that were reported from different places by independent healthcare professionals presented with similar clinical features.

Adverse drug reactions (ADRs) are a leading cause of preventable patient harm with huge socioeconomic significance. Seven contributions were received on this topic. Bassi et al. conducted a study in healthcare facilities in Nigeria where they assessed the development of ADRs in malaria patients taking artemisinin‐based combination therapy. The study consisted of two groups; patients with and without comorbidities. The findings from this study indicated that patients with comorbidities had a higher probability of developing ADRs compared to those without comorbidities. Additionally, patients with HIV/AIDS or osteoarthritis were mostly associated with the development of ADRs.

Kiguba et al. determined the prevalence of antibiotic‐associated (aa) suspected ADRs at admission and hospitalization among Ugandan patients. Previous studies reported aa‐ADRs to be extremely common which led the authors to expand on the studies and characterize the aa‐ADRs. The findings from the cohort study confirmed that aa‐ADRs were prevalent and could be as a result of patients consuming antibiotics for HIV‐associated comorbidities. At least one antibiotic was implicated in hospital‐acquired aa‐ADR, while six were associated with the development of community‐acquired aa‐ADRs.

Oshikoya et al. reported on ADRs recorded in the National Pharmacovigilance Centre in Nigeria, over a 12‐year period. Antibiotics accounted for 7.4% and fluoroquinolones for 18.7% of total ADR cases.

Awodele determined the occurrence of ADRs reported to NAFDAC Pharmacovigilance (Nigeria) from January to June 2015. The study focused on various factors pertaining to the ADRs such as the organ system affected, the completeness of the reports and the relationship between the occurrence of the ADRs and the suspected drugs. It was noted that the occurrence of ADRs was significantly high in females between 21 and 40 years of age. Antiretroviral drugs were found to contribute to the high occurrence of ADRs, with body itching being the most common. The authors suggest that more attention should be paid to HIV‐positive patients due to their susceptibility to ADRs.

A systematic review by Tegegne et al. involved a search using databases such as PubMed, Scopus, and Google Scholar to source literature on medication‐related problems and ADRs. It was found that two‐thirds and one‐third of patients developed medical‐related problems and ADRs, respectively. It was suggested that research be undertaken in order to establish mechanisms whereby to reduce the development of medical‐related problems.

Cohen et al. reported on the burden of ADRs in sub‐Saharan Africa in the era of ART. From the literature sourced using databases such as Medline, CINAHL and Scopus, antiretrovirals and antituberculosis drugs were found to be the main cause of ADRs. It was suggested that the burden of ADRs on the region should be further studied and characterized using standardized methodology.

The introduction of ART has led to a decrease in early mortality and a better quality of life in HIV‐patients. However, like any other drug, ART also results in ADRs affecting the kidneys and immune system. A review article by Ndlovu et al. evaluated the type and phenotype of drug‐induced liver injury caused by first line ART regimens. This review article also highlighted previous literature that assessed molecular mechanisms of drug‐induced liver injury, immunological responses, and biochemical pathways.

Masuka et al. investigated the reporting trends and characteristics of Individual Case Safety Reports (ICSRs) from the Zimbabwean National Pharmacovigilance System. The number of ICSRs were noted to gradually increase over time, with a significant increase observed in 2004 when public health programs and active surveillance systems were introduced in Zimbabwe. The increase in reported cases was said to be an indication that the pharmacovigilance system in Zimbabwe is functional. However, without a denominator it remains unclear whether there is in fact better reporting or more common ADRs.

Tefera et al. evaluated the off‐label drug use in pediatric patients admitted in Gondar University Referral Hospital, North‐ West Ethiopia. The off‐label use of drugs at this referral hospital was 75.8%. The highest off‐label usage was reported for antimicrobials (60.6%), followed by central nervous system drugs (14.3%). To combat this issue, the authors suggested that there should be more literature on the safety profile and effectiveness of off‐label drug use. Tefera et al. also conducted a cross‐sectional study in the Outpatient Department at the Chronic Illness Clinic of Gondor University Specialized Hospital in Ethiopia to assess the cardiovascular drug use pattern and the impact thereof on clinical outcome. Consistent with international literature, diuretic monotherapy or co‐administration with angiotensin convertase enzyme inhibitors were the most prescribed drugs in cardiovascular patients. Healthcare professionals were found to be limited to prescribing certain drugs despite there being a wide range of options.

Amagon et al. assessed the modulatory effect of co‐administration of methionine and vitamin B‐complex on antitubercular drug‐induced toxicity in patients with tuberculosis (TB). This was based on the premises that antitubercular drugs often lead to drug‐related toxicity which can be modulated by compounds such as methionine and vitamin B‐complex. Samples from the subjects (with TB) were analyzed to assess hematological, biochemical and antioxidant parameters. Methionine and vitamin B‐complex were shown to improve hepatic, renal, hematological, antioxidant indices, and adverse effects usually observed in patients with TB.

The magnitude and predictors of first‐line antiretroviral treatment failure among HIV‐infected children in Fiche and Kuyu Hospitals, Oromia Region, Ethiopia, was the topic of investigation by Grebretekle et al. In this study it was found that not only is treatment failure among childrenhigh, but that the time to faiure is very short. Treatment failure was ascribed to suboptimal adherence, lack of follow‐ups or check‐ups, initial CD4 count <50 cells/mm^3^, initial WHO stages 3 and 4. The authors suggested that more in‐depth research be conducted to determine whether disclosure plays a role in treatment failure.

In the study by Offor et al. the authors reviewed medical records of new‐borns with birth defects at a Tertiary Hospital in Southwestern Nigeria, in an attempt to decipher the teratogenic and pathologic risk factors of birth abnormalities. Down's syndrome, congenital hydrocephalus, congenital heart defect, and deformity of the digits were some of the birth defects noted. Several limitations, such as incomplete documentation, resulted in the authors not being able to find an association between maternal medications or environmental exposures with the observed abnormalities.

Mansour et al. investigated the impact of vitamin D supplementation in combination with standard treatment on pediatric patients with severe atopic dermatitis (AD), a chronic relapsing inflammatory skin disease associated with skin flares and negative effects on the patient's quality of life. The authors used serum 25(OH) D analysis and clinical assessments to measure the vitamin D concentrations and assess the severity of eczema, in the patients, respectively. The findings indicate that vitamin D supplementation might help to improve the severity of AD.

In the review article by Naidu et al. the authors investigated the role of stereological techniques (2D analysis) in assessing the testicular morphology when highly active antiretroviral therapy (HAART) was administered using a nanoparticle (NP) drug delivery system. The review also touched on NP penetration and pharmacokinetics concerning the testicular tissue and blood–testis barrier. It was suggested that in vivo studies that are conducted to evaluate testicular morphology make use of NPs conjugated with ARTs and use the stereological approach.

In a research article by Thelingwani et al. the metabolism of racemic praziquantel (PZQ) enantiomers was determined. The PZQ enantiomers which are used to treat schistosomiasis have different functions and metabolic characteristics. In vitro and in vivo analysis was used to decipher the mechanism responsible for the difference in pharmacokinetics and clearance of R‐ and S‐PQZ in the body. It was found that the enantiomers are metabolized by different enzyme proteins in the liver. The metabolism of these enantiomers determines the efficacy and safety of the drug.Therefore, the findings from this research contribute to the knowledge for use in drug development. A second contribution by Thelingwani et al. further assessed the risk of drug–drug interactions after the administration of PQZ in HIV patients who were on efavirenz or ritonavir regimes. Drug–drug interactions were found to occur when PQZ was co‐administered with efavirenz. The latter was validated by a decrease in the exposure of the enantiomers when ritonavir was co‐administered with PQZ.

Fitsum et al. determined whether the association of tamsulosin and priapism is causal. This was done using the Austin Bradford Hill Criteria for judging the evidence of causality. The authors confirmed that the association between tamsulosin and priapism is causal. Furthermore, they suggested that healthcare professionals should be cautious when prescribing tamsulosin, and that patients should be warned of the risks, due to the seriousness of the disease.

Overall, this special issue highlights the diversity and importance of pharmacological research in Africa. Many of the topics discussed in this themed issue, e.g. antibiotic resistance, while clearly applicable to the African continent also apply to many other jurisdictions, although some non‐African countries do have significant resources to mitigate such effects on patients. Opportunity costs, health prioritization and value for money are key pharmacological issues developed in this Issue and which could alert even well‐resourced countries to better value medication practices.

## AUTHOR CONTRIBUTIONS

V. Steenkamp wrote the editorial review.

## CONFLICT OF INTEREST STATEMENT

The author declares no conflict of interest.

## ETHICS STATEMENT

N/A

## PATIENT CONSENT STATEMENT

N/A

## PERMISSION TO REPRODUCE MATERIAL FROM OTHER SOURCES

No material reproduced.

## CLINICAL TRIAL REGISTRATION

N/A

## Data Availability

This is an Editorial Review, therefore the information was obtained from the accepted papers in this issue.

## References

[1] Ayele AA , Gebresillassie BM , Erku DA , et al. Prospective evaluation of ceftriaxone use in medical and emergency wards of Gondar University Referral Hospital, Ethiopia. Pharmacol Res Perspect. 2018;6(1):e00383. 29417764 10.1002/prp2.383 PMC5817827

[2] Stingl JC , Scholl C , Bosch JE , Viviani R . Genetic polymorphism of CYP2C19 and subcortical variability in the human adult brain. Transl Psychiatry. 2021;11(1):467. 34497262 10.1038/s41398-021-01591-5 PMC8426391

